# Transcriptomic landscapes of ornamental plants under salt and drought stress: key genes and pathways for tolerance

**DOI:** 10.3389/fpls.2025.1703649

**Published:** 2025-11-17

**Authors:** Alaa A Alaswad

**Affiliations:** Department of Biological Sciences, Collage of Science, University of Jeddah, Jeddah, Saudi Arabia

**Keywords:** transcriptomics, ornamental plants, abiotic stress tolerance, drought and salinity, regulatory networks

## Abstract

Salinity and drought are major ecological stresses that threaten the growth, appearance, and market value of ornamental plants. Transcriptomic studies have revealed the molecular basis of these responses, identifying pathways such as abscisic acid signaling, ion regulation, osmolyte accumulation, aquaporin-mediated water transport, antioxidant defense, and wax biosynthesis. Key transcription factor families (DREB/CBF, NAC, MYB, bZIP, WRKY, ERF) emerge as central regulators, acting alongside post-transcriptional and epitranscriptomic layers including alternative splicing, microRNAs, long non-coding RNAs, and m^6^A RNA modifications. Comparative analyses across *roses*, *chrysanthemums*, *petunias*, *irises*, and orchids reveal both conserved regulatory themes and species-specific adaptations, highlighting resilience mechanisms unique to ornamentals. This review synthesizes these insights and emphasizes their translational potential, particularly through marker-assisted selection and genome editing, to accelerate the breeding of ornamental varieties adapted to climate change.

## 1 Introduction

Ornamental plants improve the beauty of gardens, parks and homes. These plants serve the global market while supporting psychological well-being and promoting environmentally sustainable industries. As the climate change advances, drought and soil salinity problems are increasingly becoming problematic to these plants. Ornamental plants are culturally and economically important, although we have not been as well-informed about their response to environmental stress compared with major crops.

A comprehensive survey of approximately 2,600 publications from 1995 to 2025 indicates that drought stress research has been predominantly cantered on food and industrial crops, while ornamental plants have received comparatively limited scientific attention. In general, investigations into abiotic stress have largely prioritized staple crops, resulting in a significant research gap for ornamentals and other underexplored species (Nafees et al., 2019). This gap limits the development of resilience strategies for plants that are vital to cultural, aesthetic, and ecological landscapes. According to Karagüzel (2025), such exclusion means the lack of scientific discovery. The review concludes that due to the escalating climate change, physiological, molecular, and ecological multi-disciplinary approach is required to ensure the protection of ornamental plants. This gap in the body of research is important in ensuring the sustainability of the $30 billion global business based on the production of plants that are used to decorate more so with the increasing frequency of droughts.

The Omics studies enhance the understanding of the mechanisms of managing the stress of the decorative plants. Multi-omics and transcriptomic approaches map the molecular stress responses of plants to environmental stresses. In research on blue honeysuckle (Zang et al., 2025), more than 4,000 stress genes were identified and nearly 1,400 metabolites were associated with various pathways under salt stress. Multi-omics analyses of cereals and tomato (Liu et al., 2025a) identified factors and enzymes elevating drought and salinity resistance.

Recent research on plant stress responses has increasingly focused on regulatory and post-transcriptional mechanisms, highlighting the pivotal role of non-coding RNAs in stress adaptation. According to Chen et al. (2025), microRNAs, long non-coding RNAs, and circular RNAs collectively modulate the molecular networks that govern plant responses to environmental challenges. In contrast to bulk RNA-seq, single-cell transcriptomics offers a higher-resolution view of plant stress responses by uncovering regulatory programs at the individual cell level. Utilizing this approach, Liu et al. (2025b) demonstrated that root hair cells exhibit a conserved functional role in mediating salt-stress responses across different plant species.

The integration of multi-omics approaches enhances the resilience and adaptive stability of ornamental plants. This review further highlights the transcriptomic insights into salinity and drought stress responses in ornamentals, identifying key genes, signaling pathways, and regulatory mechanisms that can inform the development of climate-resilient and aesthetically robust cultivars.

## 2 Methodological advances in transcriptomics

### 2.1 High-throughput platforms and assemblies

RNA-seq remains the workhorse for ornamental species that lack complete genomes; long-read sequencing now resolves full-length isoforms and alternative splicing during salt/drought, while *de novo* assemblies combined with orthology mapping recover pathway-level signals (ABA, ion transport, ROS) and cultivar contrasts. Network-aware designs (WGCNA, module enrichment) integrate short-read profiles with proteomics/metabolomics to pinpoint candidate transporters and TF hubs.

Co-regulated modules in line with salt and drought tolerance are characterized by gene ontology, KEgg pathway enrichment, and WGCNA. These approaches together with proteomics and metabolomics enhance pathway inferences. Recent studies demonstrate through RNA-seq that chrysanthemum under salt stress exhibits altered activity in transporters and signaling pathways. In addition, defense responses and ion balance were also shared among multi-omics study of salt-stressed roses under stress spots (Ren et al., 2024).

### 2.2 Network-level and time-course designs

Time-series datasets capture early osmotic signaling through late ionic/toxic phases, while WGCNA pinpoints hub TFs and transporters predictive of tolerance. Even when demonstrated in non-ornamentals, these designs translate directly to ornamentals (e.g., time-course + WGCNA in drought studies) and are increasingly used for horticultural taxa (Wu et al., 2022).

### 2.3 Single-cell and spatial transcriptomics (emerging)

**Figure 1:**
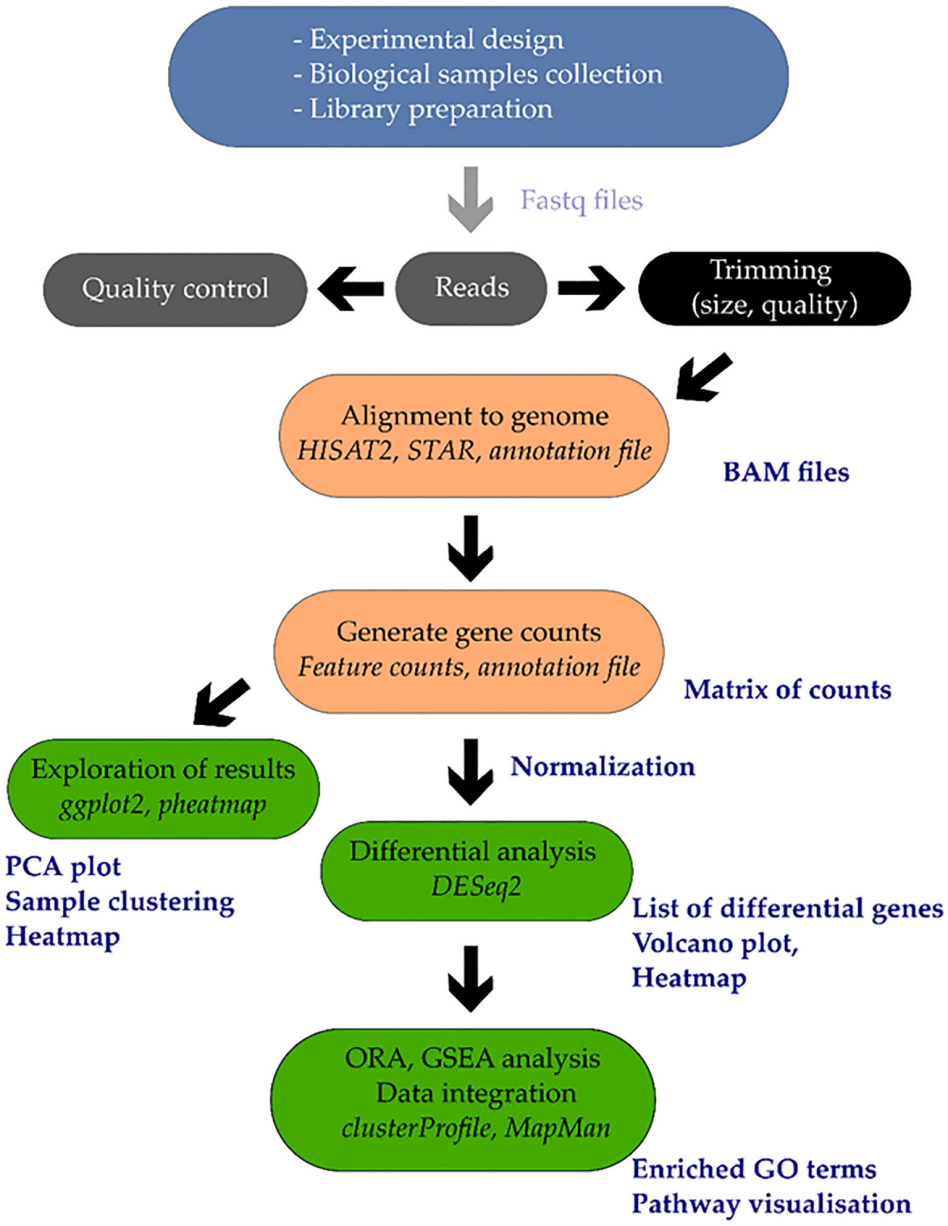
The transcriptomic data of stressed plants. Sequencing and RNA extraction are done after stress treatment. Quantification of genes occurs after the process of read processing and alignment. Analyses are differential gene expression, functional annotation using GO/KEGG and network construction using WGCNA. Cell capture and tissue mapping with single-cell and spatial transcriptomics accrue cell-specific expression that is linked with ABA signaling, aquaporins regulation, and ion exclusion during stress.

Single-cell and spatial transcriptomics are emerging tools that resolve cell-type–specific stress programs masked in bulk data, revealing root-hair and endodermal modules for aquaporin control, ABA-dependent barrier formation, and ion exclusion; these designs are now technically feasible for ornamentals with complex floral and vegetative tissues and will clarify how cell identities negotiate water and salt gradients (**Figure 1**). These methods can display expression profiles for certain cell types, like root hairs and endodermal cells, which bulk RNA-seq averages. Root cell atlases under salinity stress, such as those for cotton, have displayed how good this method is, locating ABA-related barrier creation, aquaporin changing, and ion-exclusion programs. These improvements get ready for work in ornamentals, where complex floral and vegetative tissues require cell-type-specific data to get stress adaptation (Li et al., 2024).

## 3 Core stress-responsive pathways

### 3.1 ABA signaling and interactions

**Figure 2:**
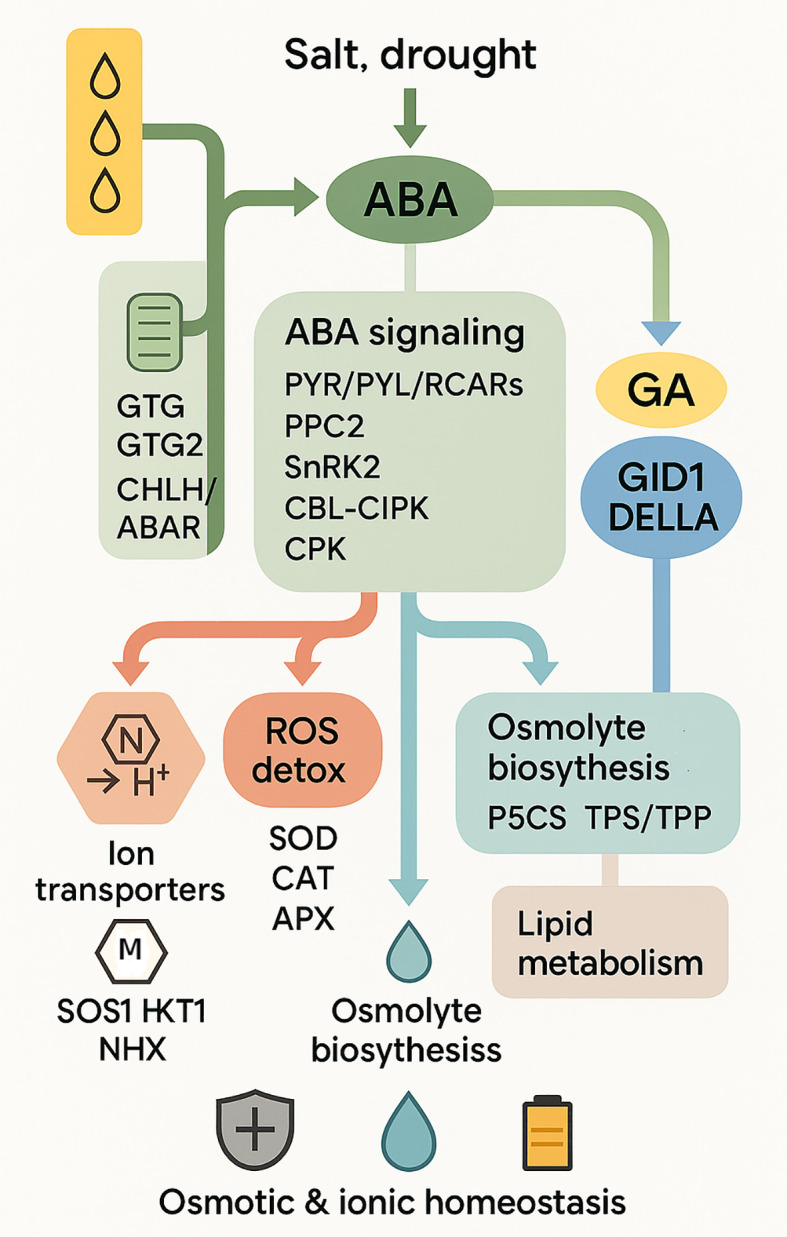
Schematic model of plant responses to salt and drought stress. The diagram illustrates stress perception in the apoplast and plastid (*GTG1, GTG2, CHLH/ABAR*), ABA signaling through receptors (*PYR/PYL/RCARs*) and kinases (*SnRK2, CBL–CIPK, CPK*, MAPK cascade, *CaM/CMLs*), and hormonal crosstalk with jasmonate (*JAZ*), gibberellin (*GA–GID1–DELLA*), and XERICO. Transcription factors (bZIP, NAC, MYB, WRKY, AP2/ERF, MPK) mediate downstream gene expression, leading to the activation of effector clusters including ion transporters (*SOS1, HKT1, NHX*, aquaporins), osmolyte biosynthesis (*P5CS, TPS/TPP*), antioxidant enzymes (SOD, CAT, APX), and lipid metabolism genes. These pathways culminate in physiological outcomes such as osmotic and ionic homeostasis, structural membrane adaptation, and maintenance of energy supply under stress conditions.

The reaction of plants to droughts and salinity relies on abscisic acid (ABA). It acts as an alert that switches defenses easily. Stomata closed down by ABA accumulation when a plant is stressed decreases water loss. Meanwhile, it prepares stress-related genes in order to allow the plant to cope with future stress. The mechanisms of this preparation have more than changes in gene activity. ABA influences alternative splicing and the way the chromatin is modified, and this enables the transcriptome to be more flexible. This allows the plants to regulate their responses in various control points. Recent studies demonstrate the importance of ABA-primed resilience. Plants that are subjected to ABA in advance may accelerate responses to stress through the rapid switching of isoforms and acclimatization of defense processes (Collin et al., 2025). ABA is not, therefore, a stress signal. It conditions plants to react more quickly and intensively on traumatizing them again. ABA normally interacts with other signals. It also connects to calcium (Ca^2+^) signaling and MAPK cascades, forming a complex interacting with tolerance. This integration ensures good coordination of osmotic adjustment, antioxidant defense and ion balance (**Figure 2**). In the case of the ornamental plants, this implies that they would endure and perform well in the varying environments thus validating ABA as a primary stress resilience regulator.

### 3.2 Ion homeostasis: the SOS1/HKT/NHX pathway

Salt at a high level is toxic to the plant cells. Plants have to control the absorption of sodium and have a balance between sodium and potassium to ensure enzyme activity and photosynthesis. Experiments with salt-stressed plants, such as chrysanthemum, demonstrate that genes of the *SOS1, HKT1, NHX* transporter groups are expressed more, and it means that they aid the plants to survive in salt (Wang et al., 2022a). *SOS1* transporter is a sodium pump that takes sodium out of the cell reducing internal sodium. The sodium is removed by *HKT1* proteins in the xylem and there is a limited amount that travels to other parts of the plant. *NHX* transporters accumulate sodium in the vacuole, and it is unable to influence the cytoplasm. These transporters cooperate to regulate ions during salt stress. This pathway interfaces with ABA signaling a core stress-hormone pathway that drives stomatal closure, transcriptional reprogramming, and ion/osmotic homeostasis under drought and salinity. ROS detoxification and accumulation of osmolytes. The *SOS1/HKT/NHX* pathway in plants ensures survival of the plants and enables them to remain and grow further as well as flower in salty environments. Therefore, *SOS1, HKT1* and *NHX* genes can be bred or edited to enable plants to be more salt resistant.

### 3.3 Osmolyte biosynthesis: proline, trehalose, and raffinose

Ornamental plants produce osmolytes such as proline, trehalose and raffinose to help plants endure drought and salinity by cushioning the structure of the cell. Transcriptomic and metabolomic studies show that water shortage or saline stress induces the expression of osmolyte synthesis genes (Renzetti et al., 2024). The *P5CS* gene family is relevant in the formation of proline. An increase in P5CS results in proline build-up during drought and salinity, which stabilize cells and redox by countering reactive oxygen species (ROS). This renders proline to be a required metabolite to resist stress. To make cells continue to work under osmotic stress, *TPS* and *TPP* regulate the level of trehalose metabolism that stabilizes proteins and membranes. Similarly, the genes of RFO pathways enhance osmoprotection and antioxidant activity. These osmolyte pathways are important to be coordinately controlled. The genes of proline, trehalose, and raffinose formation are expressed in concert with other stress-responsive genes, indicating the existence of a highly coordinated stress response. This enhances survival and retains important growth and flowering attributes that are needed in the visual and commercial markets of the ornamentals. The development of osmolytes may optimize markers and develop metabolic and mating patterns to extend the life of ornamental crops.

### 3.4 Aquaporins (PIPs/TIPs) mediate water transportation

Aquaporins, membrane proteins forming water channels, have a role in water transport, helping plants survive drought and high salt levels. Plasma membrane intrinsic proteins (*PIPs*) and tonoplast intrinsic proteins (*TIPs*) control hydraulic conductivity and water distribution in cells. Aquaporin expression and activity change under abiotic stress. In drought conditions, some *PIP* isoforms raise water absorption, whereas others cut water loss. Under high salt, *TIPs* transfer water into vacuoles to reduce osmotic stress and maintain ion balance, which supports continued photosynthesis and reproductive development even under drought or saline conditions (Byrt et al., 2023). Studies show that aquaporin control varies by cell type. Root hair and endodermal cells show different aquaporin expression when exposed to osmotic or salt stress, because of their roles in water absorption and barrier creation. Aquaporin dynamics respond to each cell type’s needs. For ornamentals, *PIP* and *TIP* control is an adaptation to keep water balance, which affects visual quality and growth. A better understanding of aquaporin control may allow for breeding or other methods to get ornamental plants that stay productive even when water conditions are poor.

### 3.5 Reactive oxygen species detoxification and redox buffering

Stresses like salinity and drought disturb plant cells, which causes the overproduction of reactive oxygen species (ROS). Large amounts of ROS can harm proteins, lipids, and nucleic acids. Plants have developed systems to detoxify ROS and use them as signals. Because of this dual role, managing ROS is very important for stress resilience.

Studies of tolerant ornamental plants show that genes for antioxidant enzymes are highly produced. These enzymes include superoxide dismutase (SOD), which changes superoxide radicals into hydrogen peroxide. Catalase (CAT) and ascorbate peroxidase (APX) then detoxify hydrogen peroxide into water and oxygen. These enzymatic defense are supported by antioxidants like flavonoids, ascorbate, and glutathione, which create a redox buffering system that stops cell damage.

Recent studies (2024–2025) suggest that ROS are regulators that coordinate stress responses (Wang et al., 2024a). ROS bursts can act as messengers in ABA and MAPK signaling, adjusting stomatal closure, ion transport, and gene expression during stress. This view is supported by ornamental transcriptomes, where antioxidant pathways coexist with ROS-mediated signaling activity.

In ornamental plants, ROS detoxification and signaling are important for survival and traits like leaf color, flower longevity, and appearance in stressful environments. By understanding these pathways, breeders and biotechnologists can equip ornamental plants to survive in environments where oxidative stress is common.

Evidence is strongest where transcriptomics aligns with functional assays in ornamentals; however, claims inferred only from Arabidopsis homologs remain provisional until validated in species like rose or chrysanthemum. A near-term translational takeaway is that antioxidant enzyme ratios (SOD: CAT : APX) can serve as robust markers for selecting stress-tolerant varieties.

### 3.6 Cuticle and wax biosynthesis

Cuticle and wax deposition increases under drought and salinity through ER-based very-long-chain fatty-acid elongation and downstream branches: alkane formation (*CER1/CER3*), primary alcohols (*FARs*), and export via *ABCG* transporters with lipid transfer proteins; coordinated upregulation of *CER1/2/3, GPATs, LTPs* thickens and re-composes epicuticular waxes, reducing transpirational water loss and surface ionic damage while preserving petal/leaf gloss and longevity (von Wettstein-Knowles, 1987). The plant cuticle is the major defense system which is mainly comprised of cutin and the waxes against environmental stressors. Many ornamental plants in response to drought and salinity up-regulate genes associated with cuticle and wax synthesis including *CER1, CER2, CER3*, glycerol-3-phosphate acyltransferase (*GPATs*) and lipid transfer proteins (*LTPs*). Cuticular wax layers are accumulated and altered by these genes and strengthens the protective barrier of leaves and petals (Wang et al., 2024a). Such an adjustment comes in handy in a few ways. A thicker and/or chemically modified wax layer lowers the loss of water, which conserves moisture in dry periods. It also offers protection against salt and oxidation injury and enhances protection to the pathogen that may gain access to the weakened tissues during stress. The cuticular integrity does play a critical role in maintaining the gloss, color, and longevity of ornamental plants by maintaining their looks, with the look of leaves and petals determining economic value in very harsh environments. Recent transcriptome-integrated wax research demonstrates that transcriptome pathways concerning wax tend to be elevated in stress tolerant varieties. In addition, the research on the epidermis implies that the amount of the wax deposition varies according to the tissue; in leaves, the structure of the surfaces is oriented on the saving of water, whereas in petals, the structure of the wax is adjusted to the maintenance of the texture and the look. Such tissue-specific change demonstrates how versatile the process of wax production is as a response to stress. Cuticular wax pathways highlight potential avenues for tolerance selection in breeding and biotechnology. On the one hand, transcriptomics can be used to target the *CER, GPAT*, or *LTP* genes, which might enhance drought and salt resistance and preserve ornamental features, ensuring that a plant will be powerful and attractive when addressing climate-related issues (Golldack et al., 2014). Evidence for wax biosynthesis genes is well supported by RNA-seq and tissue-specific studies, though functional tests remain limited. Translationally, *CER* and *GPAT* genes are promising candidates for marker-assisted breeding to improve both drought resistance and petal gloss retention.

## 4 Regulatory networks: transcription factors

We treat core pathways and their regulators as a single system: early signaling (Ca²^+^/ROS/NO/IP_3_) feeds kinase cascades (MAPKs, SnRK2s, CBL–CIPK/CPKs), which converge on TF hubs (NAC, DREB/CBF, bZIP, WRKY, MYB, ERF) that allocate transcriptional resources to ion homeostasis, hydraulic control, osmolyte production, redox buffering, and surface protection (Zhou et al., 2020). DREB/CBF and NAC transcription factor groups are important in how plants react to abiotic stresses (Golldack et al., 2014). To integrate these regulators with the core pathways, it is important to note that each TF family coordinates specific effector clusters: bZIPs-ABA-responsive-coordinate Na^+^ exclusion/K^+^ retention genes with ROS enzymes; MYB-bHLH–WD40 couples flavonoid shields with ROS control; WRKYs bridge ABA and antioxidant modules for stomatal behavior; NACs drive osmolyte production and cell wall remodeling; DREB/CBFs orchestrate osmoprotectant and antioxidant genes under dehydration; and ERFs connect ethylene signaling with cuticle and wax deposition.

They change the activity of many genes that help plants withstand drought and salinity. DREB/CBF factors are involved in the ABA-independent signaling pathway. When salt or drought stress occurs, they bind to dehydration-responsive elements in the promoters of target genes. This quickly begins processes related to osmolyte accumulation, ROS detoxification, and membrane stabilization. Studies on ornamentals show that *DREB/CBF* genes are usually more active in species that can withstand stress. The *DREB* modules are involved in stress adaptation, as displayed by their strong activation in drought-resistant petunia and chrysanthemum. *NAC* transcription factors are another big group that has diverse effects. Some *NACs* bring together stress signals, linking ABA, ROS, and ion balance. During drought in roses and irises, *NAC* members go up in number, acting as central points in Weighted Gene Co-expression Network Analysis (WGCNA) modules. *NAC* genes often appear as hubs in tolerant types based on these analyses, which means that they control entire subnetworks of protective genes. Experiments show that after increased expression, some *NACs* tested in chrysanthemum and related ornamentals displayed greater salt and drought tolerance because of increased proline accumulation and activated antioxidant protections.

In conclusion, *DREB/CBF* and *NAC* factors offer molecular targets for plant breeding and biotechnology. Their consistent appearance in stress-responsive gene networks makes them useful candidates for marker-assisted selection or genome editing. By studying these families, researchers can use master regulators to create climate-change-resistant ornamentals that keep their aesthetic qualities (Meng et al., 2022). Evidence is strongest where *NAC* overexpression has been shown to enhance osmolyte accumulation in ornamentals; by contrast, evidence based only on heterologous Arabidopsis systems is tentative. *NAC* markers linked to proline metabolism (*P5CS*) are strong candidates for early screening.

### 4.1 MYB family-ornamental validation

**Figure 3:**
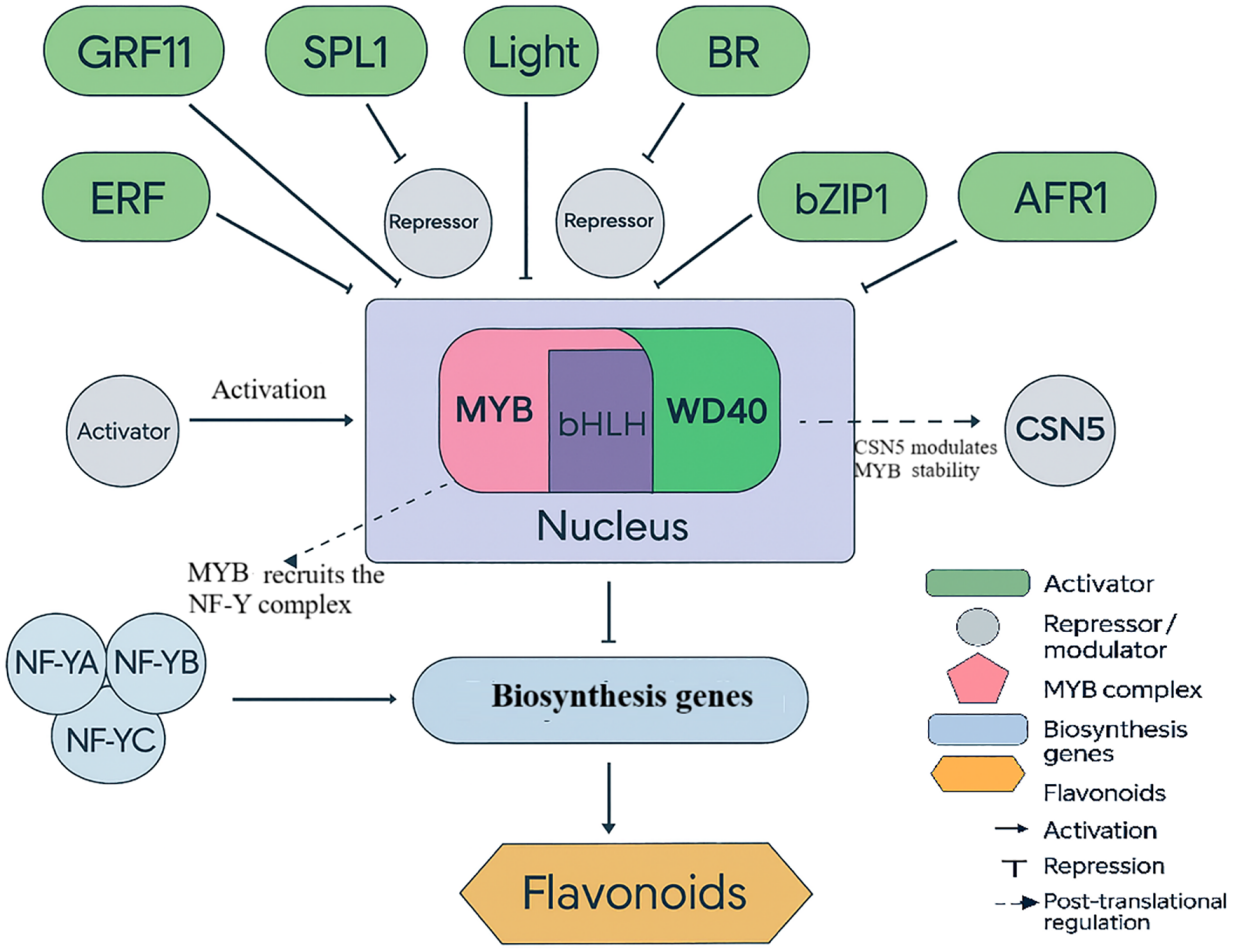
Regulation of flavonoid biosynthesis in ornamental plants. The diagram highlights the MYB–bHLH–WD40 (MYB) complex as a central hub that integrates signals from activators and repressors (including NF-Y recruitment and CSN5-mediated post-translational modulation) to control flavonoid biosynthetic genes, determining pigmentation in species such as *rose* and *chrysanthemum*.

MYB transcription factors regulate diverse pathways including flavonoid biosynthesis, ion homeostasis, and stress-induced metabolite production. For example, *PgMYB2* in *Panax ginseng* regulates dammarenediol synthase in a MeJA-dependent manner (Liu et al., 2019), illustrating how MYB proteins integrate hormonal signals with secondary metabolism - a mechanism likely mirrored in ornamentals under abiotic stress. *RcMYB8* displays the functioning of MYB transcription factors in ornamentals in roses. It has been shown to control the adaptation of plants to drought and salty soils, which is associated with improved stress tolerance under the influence of the consumption of the mentioned roles by MYB. Increased work of *RcMYB8* helps plants to be more resistant to stress. These genes contribute to water retention and ionic homeostasis under stress. Reduced *RcMYB8* expression increases susceptibility to dehydration and compromises the plant’s ability to withstand salinity and drought. *RcMYB8* protects plants by regulating such a defense gene as *RcPR5/1* and *RcP5CS1* is an enzyme producing proline. Proline defends the cells and transmits messages. *RcMYB8* is a defense-metabolic response that integrates into a single stress response (**Figure 3**). This example is important because it establishes a definite cause-and-effect correlation in an ornamental plant. Numerous MYB genes have been identified by other work under transcriptomics. Experimental validation of *RcMYB8* demonstrates its causal role in stress tolerance. This state of affairs implies that employing transcriptomic data with functional genomics can be used to identify sound breeding objectives in ornamentals (Zhang et al., 2024b).

### 4.2 Heat shock and MYB complexes in chrysanthemum

Heat shock proteins (HSPs) function as molecular chaperones that stabilize proteins and membranes under abiotic stress. For instance, *TaHSP17.4*, interacting with *TaHOP* in wheat, was shown to enhance stress tolerance (Wang et al., 2023), reinforcing the role of small HSPs as crucial modulators of plant resilience. Recently, Wang et al. (2024b) discussed a regulatory system in *chrysanthemum* that connects heat-shock signaling with how well it tolerates salt. Their work suggests that a *CmHSFA4-CmMYBS3–TPL* protein group acts as a key regulator of ionic balance when plants are exposed to salt (Feng et al., 2020). This group improves tolerance by lowering the expression of *CmMYB121*, a transcription factor linked to salt sensitivity. By lowering *CmMYB121* levels, the group activates ion transport and protective pathways, helping to maintain Na^+^ and K^+^ balance during stress. This finding matters for a few reasons. First, it implies that heat-shock factors (HSFs) are not just for thermal stress responses but are also part of abiotic stress responses through interactions with transcription factors like MYBs. Second, this repression mechanism shows how plants control gene expression by turning on protective genes and turning off negative regulators. Last, by detailing the interactions between *CmHSFA4, CmMYBS3*, and the co-repressor TPL, the study gives a specific molecular target for breeding or genome editing to raise salt tolerance in *chrysanthemum*, a common ornamental crop (**Table 1**).

**Table 1:** Validated candidate genes and transcription factors in ornamentals.

Gene/TF	Species	Function in stress tolerance	Evidence (overexpression, RNAi, etc.)	Reference
*RcMYB8 (R2R3-MYB*)	Rose (*Rosa chinensis*)	Enhances salinity & drought tolerance; regulates PR and proline (RcPR5/1, RcP5CS1)	OE in rose ↑ tolerance; VIGS/silencing ↓ tolerance	(Zhang et al., 2024b)
*RcbHLH59*	Rose (*Rosa chinensis*)	Improves salinity tolerance via Na^+^/K^+^ balance and callose deposition (RcPRs module)	OE in rose ↑ salt tolerance	(Su et al., 2023)
*DgNAC1 (NAC TF)*	*Chrysanthemum*	Positive regulator of salt stress responses	OE (heterologous) enhances salt tolerance; regulates stress-responsive genes	(Wang et al., 2017)
*CcSOS1* (PM Na^+^/H^+^ antiporter)	*Chrysanthemum crassum*/*C. morifolium* ‘Jinba’	Improves ion homeostasis under salinity	OE of CcSOS1 improved salinity tolerance in ‘Jinba’	(Guan et al., 2017)
*DgMBF1* (Multiprotein Bridging Factor 1)	*Chrysanthemum*	Enhances salt tolerance, likely via transcriptional co-activation of stress genes	OE in chrysanthemum ↑ salt tolerance	(Zhao et al., 2019)
*ZPT2-3* (TFIIIA-type zinc finger)	*Petunia ×hybrida*	Increases drought (and cold) tolerance	OE in petunia ↑ drought/cold tolerance	(Xu et al., 2008)
*PhDREB1* → *PhZFP1* → *PhGolS1* cascade	*Petunia ×hybrida*	DREB-centered pathway promoting osmolyte (galactinol) synthesis and stress resilience	Functional cascade dissection; OE/perturbation in petunia	(Zhang et al., 2023a)
*IgWRKY50/IgWRKY32* (WRKY TFs)	*Iris germanica*	Positive regulation of drought responses; ABA/heat inducible	OE in Arabidopsis ↑ drought tolerance; strong induction under PEG	(Zhang et al., 2022)
*DnWRKY11* (WRKY TF)	*Dendrobium nobile* (orchid)	Enhances drought & salt tolerance	OE in tobacco ↑ drought/salt tolerance (functional validation)	(Xu et al., 2014)
*DnMSI1* (chromatin/RNA-associated)	*Dendrobium nobile* (orchid)	Negative regulator of salt tolerance	OE in Arabidopsis reduced NaCl tolerance (sensitizing plants)	(Cui et al., 2022)

↑, upregulation; ↓, downregulation.

### 4.3 WRKY, bZIP, ERF, bHLH and other transcription factors

**Figure 4:**
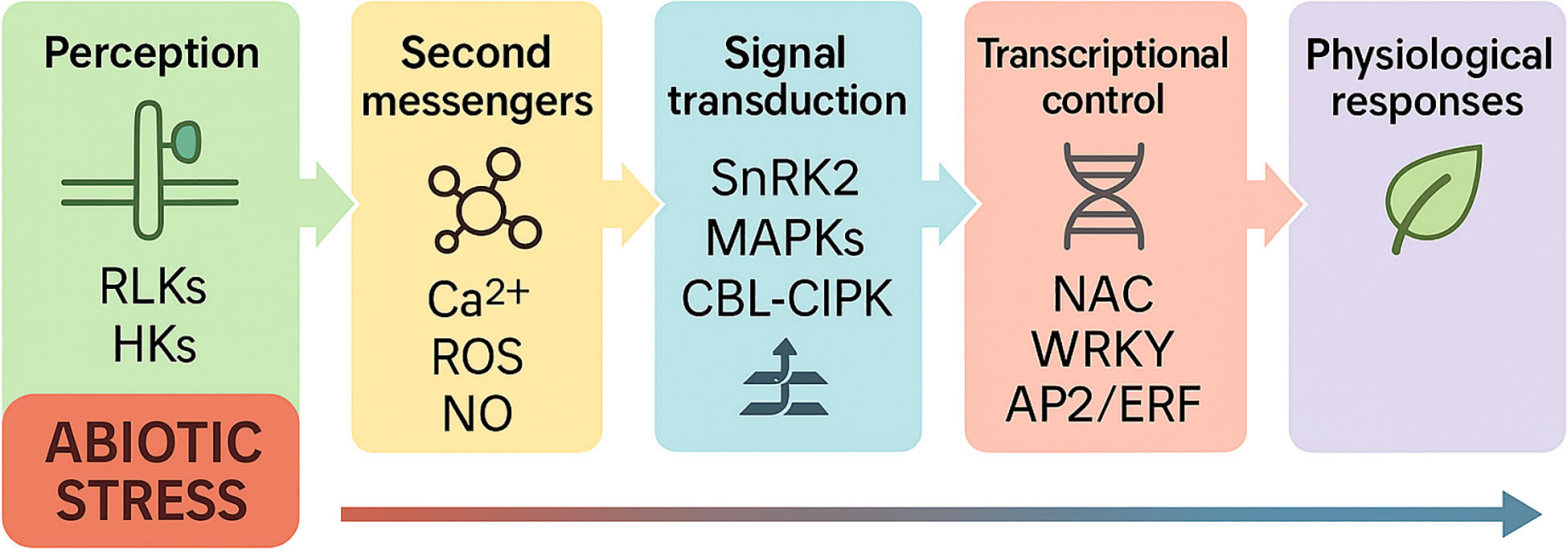
Schematic model of plant responses to abiotic stress. Abiotic stress is perceived by membrane receptors such as *RLKs* and *HKs*, which initiate intracellular signaling. This activates second messengers including Ca²^+^, ROS, NO, cAMP/cGMP, and IP_3_, which relay signals to downstream kinase cascades (*SnRK2, MAPKs, CBL–CIPK, CaM/CMLs*). These kinases in turn activate transcription factors such as NAC, WRKY, and AP2/ERF, which regulate the expression of stress-responsive genes. The coordinated transcriptional reprogramming drives physiological responses including ion transport (e.g., *SOS1, HKT1, NHX*), osmolyte accumulation, aquaporin regulation, and antioxidant defense, ultimately promoting osmotic adjustment, ionic balance, and stress tolerance under drought, salinity, and heat conditions.

Beyond DREB and NAC, several transcription-factor families-WRKY, bZIP, ERF, bHLH, TCP, and MYB-act as key integrators of the abiotic-stress network depicted in **Figure 4**. In our model, stress is perceived at the membrane and converted into second messengers (Ca²^+^ spikes, ROS, cAMP/cGMP, NO, IP_3_), which feed into signal-transduction modules (MAPKKK→MAPKK→MAPK, SnRK2, CBL–CIPK/CPK, CaM/CMLs with PP2C control). These kinase cascades converge on transcription factors that reprogram stress-responsive genes, ultimately driving ionic/osmotic balance, membrane stability, and growth adjustment.

WRKYs exemplify this integration. For instance, *IlWRKY22* in *Iris laevigata* is induced by salt and drought and enhances tolerance by boosting antioxidant capacity, stabilizing ROS levels, and modulating ABA-responsive stomatal genes-positioning WRKYs as effective relays between early stress signaling and downstream adaptation. bZIP factors, many of which are ABA-responsive, coordinate ion‐homeostasis genes (e.g., Na^+^ exclusion, K^+^ retention) with antioxidant enzymes so that ion transport and ROS detoxification proceed in concert (Sun et al., 2021). TCP proteins-classically linked to organ growth-are recruited under stress in chrysanthemum to fine-tune growth–defense trade-offs, illustrating how developmental regulators are repurposed under adversity.

Together with MYB, ERF, and bHLH families, these factors form combinatorial control points. ERFs connect ethylene and redox/osmotic programs; bHLHs frequently partner with MYBs to regulate flavonoid biosynthesis, influencing both pigmentation and protection from oxidative damage; and MYBs are consistently enriched in *rose* and *chrysanthemum* transcriptomes during stress. Overall, the TF layer operates as a systems hub linking hormonal crosstalk (ABA/ethylene), redox buffering, and ion regulation to the expression of protective genes-providing tractable targets for breeding and genome editing to enhance stress resilience without compromising ornamental quality (Wang et al., 2024c).

Functional genetics in chrysanthemum confirms WRKY roles in stomatal and antioxidant control, but broader cross-species validation is limited. WRKY hubs combined with ABA-responsive promoters offer actionable targets for breeding drought-adapted ornamentals.

## 5 Post-transcriptional and epitranscriptomic regulation

### 5.1 Alternative splicing as a dynamic stress-response mechanism

Alternative splicing (AS) is now a key part of how genes are controlled when plants face stress, giving the transcriptome more range. Many stress-related genes generate multiple isoforms rather than a single transcript, and these isoforms can fulfill different regulatory or functional roles during stress responses. When there’s salt or not enough water, plants change these transcript versions. This helps them adjust signaling, ion movement, and how they use energy, in ways that normal gene expression can’t. Current work shows that AS often relates to ABA signaling, which strengthens the hormone’s main role in managing tolerance to drought and salt levels (Alhabsi et al., 2025). Alternative splicing (AS) of ABA-responsive transcription factors and transporters can alter protein activity, subcellular localization, or interaction capacity, thereby enabling plants to rapidly adjust as stress levels fluctuate. Recent advances in long-read sequencing platforms, such as PacBio and Oxford Nanopore, are transforming our understanding of AS. Unlike short-read approaches, which often miss complex isoforms, long-read sequencing can capture full-length transcripts and resolve novel splice variants. Early applications in stressed leaves and roots have already identified previously undetected isoforms. This is particularly relevant for ornamentals, many of which lack high-quality reference genomes, making isoform-level resolution difficult. Long-read transcriptomics now offers the ability to detect AS events in key regulators, including ion transporters, aquaporins, and transcription factors, thereby providing new molecular entry points for resilience breeding. Far from being a background process, AS constitutes a dynamic regulatory mechanism that enables ornamental species to withstand salinity and drought stress while maintaining growth, pigmentation, and flowering.

### 5.2 microRNAs regulating stress-responsive genes

MicroRNAs (miRNAs) play a critical role in plant responses to stress through regulation of gene expression. Different miRNAs vary in drought or high salt, and the targets include factors and enzymes that regulate stress responses, including oxidative stress, hormone signaling and ion balance. As an example, miR398 regulates *Cu/Zn*-superoxide dismutases (*CSD1* and *CSD2*), the enzymes eliminating reactive oxygen species (ROS). The other example is miR169 that regulates *NF-YA* transcription factors that are involved in ABA-related drought responses with fast changes in stress signaling. It has been demonstrated that miRNA functions vary among plant species. Indicatively, the fluctuations in miR398 expression are also dependent on the timing of the stress exposure, which assists the plants to conserve resources and to protect themselves in a suitable manner (Nai et al., 2023). The MiRNAs provide specific manipulation of between and stress tolerance of ornamentals. By modulating the expression of critical stress-responsive genes, plants are able to maintain vegetative growth, pigment stability, and flowering even under adverse environmental conditions. The identification of new stress-responsive miRNAs such as degradome sequencing and sRNA-seq can enhance breeding of ornamental types with the ability to cope with adverse conditions better (**Table 2**).

**Table 2:** Post-transcriptional regulators linked to stress tolerance in ornamentals.

Regulator (miRNA/lncRNA/AS)	Target gene/pathway	Stress type	Species	Reference
miR398	Targets *CSD1/2*(*Cu/Zn*-*superoxide dismutase*); modulates ROS detoxification	Salt, drought	*Petunia ×hybrida*	RNA-seq + degradome studies (validated in Solanaceae)
miR169	Targets *NF-YA* transcription factors; regulates ABA-responsive genes	Drought	*Rosa chinensis*, *Petunia*	RNA-seq + qRT-PCR
miR164	Targets *NAC* TFs; influences root and leaf stress responses	Salt	*Chrysanthemum morifolium*	Transcriptomic analysis
lncRNA-XLOC-123 (rose)	Predicted interaction with *miR156/SPL* module (development + stress signaling)	Drought	*Rosa chinensis*	Full-length transcriptome study
lncRNA-CIL1 (chrysanthemum)	Interacts with ABA signaling and ion transport genes	Salt	*Chrysanthemum lavandulifolium*	RNA-seq + functional annotation
AS events (intron retention, exon skipping)	Affect *HSPs*, TFs (*MYB, WRKY*) and metabolic enzymes	Drought	*Iris germanica*	RNA-seq (PEG-induced drought)
AS of *P5CS* (Δ-1-pyrroline-5-carboxylate synthase)	Alters osmolyte biosynthesis (proline accumulation)	Salt, drought	*Petunia* and *Orchid* species	RNA-seq comparative studies

Many ornamental post-transcriptional regulators are still annotated from *RNA-seq + degradome* evidence; only some have direct experimental validation (e.g., miRNA–target cleavage, AS detection by Iso-Seq).

### 5.3 Long non-coding RNAs as stress modulators

**Figure 5:**
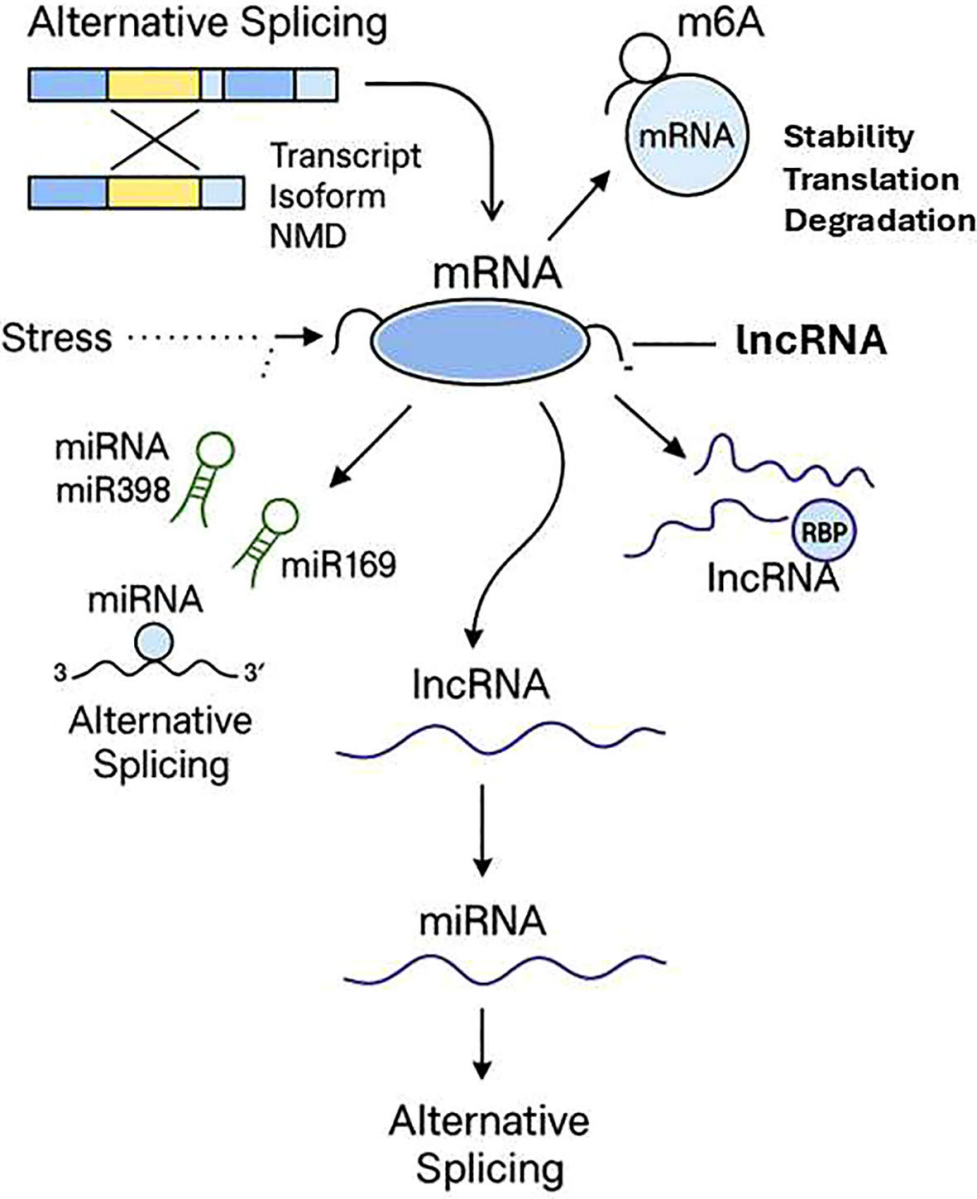
Epi- and post-transcriptional stress-response regulation. Thematic diagram of important strata of RNA-based regulation in stress. Alternative splicing produces several transcript isoforms that can be degraded by Nonsense-Mediated mRNA Decay (NMD) or transcripts degraded by miRNAs (e.g., miR398, miR169), or transcripts repressed by translation. Long noncoding RNAs (lncRNAs) regulate expression by interacting with mRNAs and RNA-binding proteins (RBPs), as well as through the splicing, stability, translation, and degradation modulated by m^6^A RNA methylation. Together, these post-transcriptional and epitranscriptomic processes refine gene expression of stress response.

In stressful situations, long non-coding RNAs (lncRNAs) have been shown to be at the center of genes regulation in plants. LncRNAs are not similar to coding genes because they can interact with transcription factors and microRNAs (miRNAs) and with stress-responsive genes. Some lncRNAs help to signal in case of drought and salt stress (Chen et al., 2022). An example is that certain lncRNAs serve as molecular sponges in that they bind to stress-associated miRNAs, such as miR398 or miR169. This binding influences the interaction of such miRNAs with their targets, and the plant changes its response to stress. This association helps the lncRNAs to avoid being suppressed by miRNA on their target genes. Other lncRNAs also interact with transcription factors or chromatin-modifying complexes and regulate the expression of genes and allow responding to stress with a more subtle manner (Huang et al., 2020) (**Figure 5**). The relations between lncRNAs and genes can be revealed with the help of co-expression analysis and modeling methods (Zhang et al., 2024a). Originally introduced in research on Arabidopsis and rice, these methods are being applied more and more to ornamental plants as reference genomes and transcriptomic information become more readily available. Research of lncRNAs in ornamental plants is especially useful because it is generally species- or tissue-specific and therefore can influence adaptations in leaves, petals or roots. This particularity may pose considerable consequences to the plant in terms of coping with stress. The discovery of these regulatory molecules can offer new molecular targets to researchers which can further be used in breeding or genetic engineering of stress resistant ornamental plants.

### 5.4 Epitranscriptomics (m^6^ and beyond)

Epitranscriptomics, which looks at chemical changes in RNA, has recently become important in plant stress studies. *N^6^-*methyladenosine (m^6^) is the most common and important of these changes. It affects RNA activity quickly and can be changed back, reacting strongly to what’s happening around it. The m^6^ system includes enzymes that add the mark (writers), proteins that read and understand it (readers), and enzymes that remove it (erasers). In plants, regulators like *FIO1* (a writer) and *ECT8* (a reader) affect how plants react to drought and salt. By changing certain mRNAs, m^6^ can change how stable they are, how they are spliced, how they leave the nucleus, or how well they are translated. This helps plants change gene expression quickly when stressed (Cai et al., 2024). New work shows that m^6^ levels change with salt and drought stress. This makes RNA modification a quick way to adjust along with transcription and post-transcriptional regulation. Plants can use this control to adjust stress-related pathways without needing new transcription, which matters when the environment changes fast. Epitranscriptomic regulation could be very useful for ornamental plants, where they need to be tough but also flower and have good color. Because m^6^ can affect many gene networks, like those for hormone signals, ion transport, and antioxidant defenses, it could act as a control panel. This helps balance being able to handle stress with growth and how the plant looks. As better sequencing methods are used more, studying m^6^ and other RNA changes in ornamentals might show new ways they are regulated. This could give breeders and biotechnologists new ways to create ornamental species that can handle climate change.

## 6 Species spotlights (ornamentals)

Across *rose*, *chrysanthemum*, *petunia*, *iris*, and *orchids*, three themes recur-(i) ion-homeostasis triad (*SOS1/HKT1/NHX*) with tissue-specific aquaporins, (ii) redox buffering tightly coupled to ABA-responsive bZIP/WRKY modules, and (iii) osmolyte metabolism (*P5CS; TPS/TPP*) coordinated by NAC/DREB-all overlaid by species-specific ornamentation programs (e.g., MYB–bHLH–WD40 control of flavonoids).” Then, for each species subsection, keep only one or two “what’s unique here” sentences (e.g., rose: flavonoid/ion cross-talk; *chrysanthemum*: *HSFA4–MYBS3–TPL*; *petunia*: time-phased drought program), and link back to the comparative themes to avoid re-listing generic pathways.

### 6.1 Rose (*Rosa* spp.)

Roses, well-known ornamental plants, are starting to show their molecular methods for handling abiotic stress using multi-omics studies. Besides the known role of *RcMYB8* in managing drought and salt tolerance, new transcriptomic and metabolomic studies under salt stress have indicated that rose hardiness relies on the interaction of full regulatory networks (Ren et al., 2024). In rose, a distinguishing feature is the flavonoid/ion crosstalk, where MYB–bHLH–WD40 complexes regulate both pigment biosynthesis and redox balance, reinforcing the comparative theme of flavonoid-linked stress adaptation.

Important pathways keep appearing across these data sets. Genes tied to ion transport are upregulated, helping to keep a good Na^+^/K^+^ balance in stressed tissues. Concurrently, phenylpropanoid and defense pathways are activated, leading to the synthesis of secondary metabolites that both reinforce structural barriers and mitigate oxidative damage. In parallel, genes associated with proline and soluble sugar metabolism support osmotic adjustment and facilitate stress recovery.

**Figure 6:**
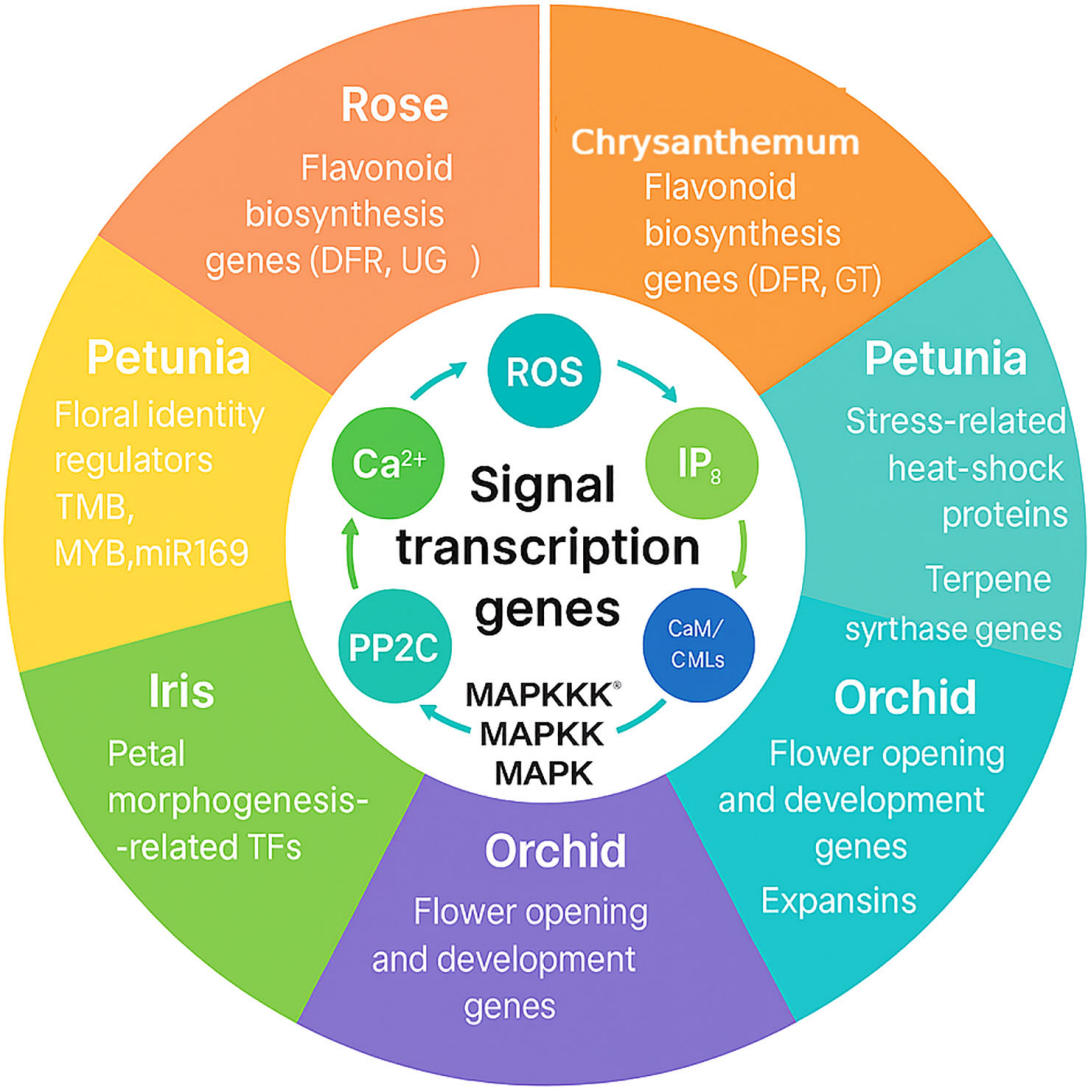
Transcriptomic case studies in ornamental species. The circular diagram links conserved signaling and transcriptional modules-Ca²^+^, ROS, IP_3_, *CaM/CMLs*, *PP2C*, and the *MAPKKK→MAPKK→MAPK* cascade-to species-specific themes identified by RNA-seq. Rose: flavonoid biosynthesis genes (*DFR, UGT*) regulated by MYB/bHLH factors. Chrysanthemum: flavonoid biosynthesis genes (*DFR, GT*). Petunia: floral identity regulators (TMB, MYB, miR169) and stress-related genes including heat-shock proteins and terpene synthases. Iris: petal morphogenesis–related transcription factors. Orchid: flower opening and developmental genes, including *expansins*. The figure highlights how conserved signaling hubs integrate with lineage-specific transcriptional programs to define ornamental traits.

These results emphasize the network-level mix of reactions. Instead of depending on single genes, hardy rose types manage a broad molecular response in which ion regulation, antioxidant action, and osmoprotection are connected. This systems-level hardiness makes sure that stress reactions are balanced, letting roses keep their ornamental value-flowering, color, and fragrance-even under salty conditions (**Figure 6**).

For breeders and biotechnologists, this gives a key lesson: aiming for full pathways or regulatory pieces, instead of single spots, might give the best methods for making stress-hardy roses that keep their commercial and aesthetic appeal.

### 6.2 Chrysanthemum

*Chrysanthemum* is a popular ornamental plant whose example is worth studying the resistance of plants to abiotic stress. Research has developed a repository of potential genes, regulatory RNAs and tolerance-related pathways in studies over the last ten years that participated transcriptomic and small RNA profiling under salt and drought conditions. According to these studies, the processes of antioxidant systems regulation, ion movement proteins, and osmolyte formation are activated and assist chrysanthemum with adaptation (Liu et al., 2022). In addition, miRNA-degradome assays have identified small RNAs, which modulate stress signals. Certain drought- and salt-relevant miRNAs were observed to regulate transcriptional or ROS-cleaning enzymes and established a control system involving both transcriptional and post-transcriptional responses. Greater understanding has been provided by functional genetics (Liu et al., 2020). Consider the *HSFA4-MYBS3-TPL* complex, which inhibits salt tolerance, and families of transcription factors such as DOF and TCP that modulate routes of ABA and ion homeostasis. These findings inform us that *chrysanthemum* can be used to learn about the complicated gene networks rather than the individual-gene responses. Work has also demonstrated that rootstock heterografting can increase salt tolerance, showing that chrysanthemum resilience is not restricted to genetic determinants but can be improved through cultivation practices including grafting and controlled-environment systems such as hydroponics. Such a combination between molecular and applied workplaces chrysanthemum at a central position in ornamental biology of stress, as not only a useful crop, but also as a paradigm of how control levels in multiple ways contribute to resilience. Chrysanthemum breeders and researchers make one understand that transcriptomic, genetic and growing approaches should be combined to produce ornamentals in unfavorable environments. *Chrysanthemum* uniquely features the *HSFA4–MYBS3–TPL* module that suppresses salt tolerance, highlighting how species-specific repressors intersect with the conserved ABA/ion homeostasis theme.

### 6.3 Petunia

*Petunia × hybrida* is a useful ornamental model for examining plant responses to drought across time. RNA-seq studies have indicated that petunia adapts to water stress in stages (Park et al., 2021). In the beginning of drought, ABA signaling and ROS processes start early, causing stomatal closure, antioxidant activity, and stress-related transcription factors that prep the plant for survival. Later stages involve shifts in metabolism, like changes in carbohydrate, amino acid, and secondary metabolite pathways, helping growth and reproduction continue when water is limited.

These molecular results match breeding and field data, making petunia particularly valuable. Broad analyses have found thousands of differentially expressed genes (DEGs) during water deficits, backing the idea that drought tolerance involves many genes. Both floral and vegetative traits are affected, showing that petunia’s resilience comes from a broad network of coordinated responses, not one single factor.

Horticulturally, petunia offers a concrete case of how genomics and breeding can be related. Knowledge of ABA signaling, ROS control, and later metabolic changes gives specific targets for marker-assisted selection. Transcriptome data sets also help in designing cultivars with better drought resistance. Petunia’s popularity shows the importance of these results, linking molecular stress biology with traits key to growers and consumers. Petunia stress responses are notable for their time-phased drought program, aligning with the universal osmolyte/ROS buffering themes but distinguished by temporal layering of gene activation.

### 6.4 Iris and other ornamentals

The Omics research is already starting to describe how *Iris* plant survives under drought and salt conditions. Transcriptomic evidence demonstrates that WRKY and bHLH families of transcription factors control stress response webs. These transcription factors seem to tune antioxidant defense, hormone signaling and osmotic adjustment, as well as enabling iris plants to threshold upon altered water conditions. There are types that regulate the ions in a manner reminiscent of that in halophytes, such as enhanced Na+ uptake and K+ retention, implying an adaptive mechanism that permits iris to maintain ion equilibrium in presence of salt (Guo et al., 2024). This is assisted by work on other ornamentals. Transcriptome and metabolome studies of Rhododendron emphasize Na^+^/Ca^2 +^ in salt tolerance. In lily (*Lilium* spp.), stress adaptation appears to be mediated by interactions of hormones with ABA, ethylene, and jasmonic acid. The implication of these results is that ornamental plants have resilience not due to individual pathways but rather because of regulatory systems. Essentially, *iris*, *rhododendron* and *lily* depict how ornamentals adopt similar and different strategies to fight salt and drought. They also suggest that cross-group comparative studies are required within ornamental groups. Such research may discover regulators and certain adaptations that might be helpful during the breeding process. Using this, researchers can optimize the resources in order to develop the ornamental to flourish in the challenging environments (**Table 3**).

**Table 3:** Summary of transcriptomic studies on ornamental plants under salt and drought stress.bottom of form.

Species	Stress type	Tissue	Platform	No. of DEGs	Key pathways identified	Reference
*Rosa chinensis*	Salt	Leaves & roots	Illumina RNA-seq	NR	ABA signaling, plant hormone signal transduction, glutathione metabolism	(Tian et al., 2018)
*Rosa chinensis*	Drought	Roots & leaves	Full-length (PacBio SMRT); RNA-seq validation	NR	Photosynthesis, ABA signaling, TFs (MYB/WRKY/NAC), ROS detox	(Lin et al., 2022)
*Chrysanthemum lavandulifolium*	Salt	Seedling leaves	DGE (Illumina HiSeq)	2,254	Ion transport (*HKT/NHX/AKT*), ROS scavenging, flavonoid & trehalose metabolism, ABA pathway	(Huang et al., 2019)
*Petunia ×hybrida*	Drought (water deficit)	Leaves	Illumina RNA-seq	195 (day 1); 6,417 (day 3)	Antioxidant enzymes (*APX/CAT/SOD/GST*), sulfur metabolism, ABA/ET/JA signaling, AP2/ERF & bZIP TFs	(Park et al., 2021)
*Iris germanica* (‘Little Dream’)	Drought (PEG-6000)	Leaves & rhizomes	Illumina RNA-seq	7,849 (leaf); 24,127 (rhizome)	Photosynthesis, hormone signaling, starch/sucrose metabolism, secondary metabolites; HSPs/TFs/ROS	(Zhang et al., 2021a)
*Iris lactea* var. *chinensis*	Drought (withholding water & rehydration)	Leaves	Illumina RNA-seq	1,187 (T/CK); 275 (R/T); 865 (R/CK)	Plant–pathogen interaction, α-linolenic acid metabolism, circadian rhythm, ABC transporters, arginine/proline metabolism	(Zhang et al., 2023b)
*Dendrobium sinense* (orchid)	Drought	Leaves (with metabolomics)	RNA-seq + metabolomics	NR	Purine metabolism, phenylpropanoid & antioxidant pathways (integrated changes under drought)	(Zhang et al., 2021a)

NR, not reported explicitly in the accessible text; the papers still detail differentially expressed pathways and gene families.

## 7 Translational applications

### 7.1 Candidate genes and markers for selection

The work of gene functioning in ornamental plants has identified genes which would be usable as molecular markers. An example is that *RcMYB8* helps the roses to endure drought and salt, and the *CmHSFA4-MYBS3* family helps chrysanthemums to survive the salt by suppressing negative regulators. According to Zhang et al. (2024b), such genes might enable the development of markers, and with their help, breeders could trace the resilience features of breeding populations. The weighted Gene Co-expression Network Analysis (WGCNA) has been applied to determine hub genes, which are critical in the response of the plant to stress. These key genes unite the signals of pathways which are related to ion transport, ABA signaling and ROS detoxification and are therefore useful multi-gene markers. The fact that hub genes are resilient at the network level can enhance predictions in terms of choosing complex stress tolerance. Application of marker-assisted selection (MAS) is widespread in primary crops, and scientists are evaluating the techniques on ornamental plants. Studies of cereals and legumes reveal that the use of multiple markers in breeding enhances the development of resilient cultivars for field performance. The same may happen to ornamental plants, where markers of transcriptomic data are applied to the breeding plans (**Table 4**). This will be positive in the ornamental plant industry. By targeting stress-response genes and regulatory hubs, breeders can generate climate-resilient varieties while preserving key ornamental qualities such as color, form, and flowering. Transcriptomics unites both molecular studies and practical horticulture where scientific data are bridged with what is desired by growers and markets.

**Table 4:** Emerging technologies in ornamental transcriptomics.

Technology	Application	Example in ornamentals	Future potential
RNA-Seq (Illumina short-read)	Global transcriptome profiling, DEG analysis	*Rose, chrysanthemum, petunia* drought/salt studies	Standardized reference datasets, integration with pan-transcriptomes
Full-length Iso-Seq (PacBio/ONT)	Alternative splicing, isoform discovery, lncRNA annotation	*Rosa chinensis* drought transcriptome	More accurate gene models, improved reference genomes for ornamentals
Single-cell RNA-Seq (scRNA-seq)	Cell-type–specific transcriptional responses to stress	Early trials in orchid tissues	Mapping cell-specific stress responses in complex floral organs
sRNA-Seq (miRNA profiling)	Identifying stress-regulatory miRNAs and targets	miR398, miR169 characterized in *petunia* and *rose*	miRNA-based biomarkers for stress tolerance breeding
WGCNA & Co-expression Networks	Module detection, hub gene identification	*Petunia* drought networks; *chrysanthemum* salt modules	Integration with GWAS/phenomics for trait prediction
Epitranscriptome Mapping (m^6^-seq, Nanopore direct RNA)	Detection of mRNA modifications affecting stability/translation	Exploratory studies in orchids	Novel layer of regulation; epitranscriptomic breeding targets
Multi-omics Integration (RNA-Seq + metabolomics/proteomics)	Linking transcripts to metabolites/proteins under stress	*Dendrobium* drought stress transcriptome–metabolome study	Systems biology approaches for ornamental trait improvement
CRISPR-based functional transcriptomics (CRISPRi/a screens)	Validating candidate stress genes by targeted knockdown/activation	Conceptual in ornamentals; tested in petunia for gene editing	High-throughput functional validation of candidate genes and TFs

### 7.2 Genome editing and functional validation

The CRISPR-mediated genome editing has ushered into quicker approaches to make up gene action and to enhance excellence to the aesthetic plants. CRISPR was initially applied largely to model plants and major crops but currently performs to a level of competence in *chrysanthemum*. Scientists have modified such genes as *CmPDS* and *CmTGA1*. These changes demonstrate that polyploid ornamentals, previously thought to be difficult to edit, can in fact be engineered (Chen et al., 2024). A dedicated CRISPR resource and database of *chrysanthemum* simplifies this process by providing a researcher with a convenient mechanism of designing and trying edits at the gene level. The system allows fast validation of transcriptome-nominated targets in RNA-seq, WGCNA or small RNA profiling and associates gene discovery to trait enhancement. In addition to early edits, CRISPR has the ability to add features to cultivars. It allows alteration of genes that control environmental tolerance without altering major traits such as color, shape or smell of flowers. Base editing and CRISPR interference (CRISPRi) and CRISPR activation (CRISPRa) also broaden the opportunities, with the ability to modify gene expression or make a single-nucleotide modification. This has the potential to enhance resilience to stress with only a little genome alteration. In the case of ornamentals, breeding is prolonged, and trait choice is complicated, genome editing provides appreciated capabilities. It accelerates validation of transcriptomic data and provides breeders with a way to engineer plants to resist stress that enables ornamental plants to remain appealing and adapted to environmental stress.

### 7.3 Use of transcriptomic data to guide genome editing

Transcriptomic data are no longer restricted to explanation of gene expression patterns but are now being used as next of direction in functional genomics as well as accuracy breeding. Transcriptome analyses can reveal candidate targets to the genome editing tools including CRISPR/Cas9, CRISPR interference/activation (CRISPRi/a), and base editors by identifying key stress-responsive genes and regulatory hubs (Wang et al., 2022b). As an example, salinity and drought stress can cause a preference in the selection of loci to edit by the differential expression of ion transporters genes, antioxidant enzymes, or transcription factors such as *NACs* and *DREBs*(An et al., 2020; Saleem et al., 2025). After validation, specific mutations-be they gene deletions, promoter changes, or point mutations- could be used to increase stress tolerance in ornamentals without affecting the desirable floral or aesthetic characteristics. These applications take the field out of the descriptive realm of omics into realistic molecular breeding and has allowed researchers to create ornamentals that can be both tough and beautiful.

### 7.4 Incorporating into breeding plans and sustainable urban horticulture

The true worth of transcriptomic results is its applicability to breeding programs. Ornamental breeding in plants typically focuses on such characteristics of the plant as the color, scent and form of flowers. However, the characteristics such as climate hardiness and resource utilization are becoming significant with the increasing number of environmental problems (Cai et al., 2021; Deng et al., 2021). Transcriptomics may accelerate the marker-assisted and genomic selection by providing molecular markers related to stress endurance, bloom period, or pigment development. Combined with genome editing, this will allow the breeders to reduce the time spent on breeding and introduce hardiness without sacrificing ornamental traits (Wu et al., 2023). Transcriptomic applications, in addition to breeding programs are connected to the concept of sustainable urban horticulture. The foundation of green infrastructure such as parks, rooftop gardens, and vertical greening systems is made of ornamental plants. Such plants are forced to contend with a lack of water and heat and salted soil. Researchers can take urban sustainability a step further by modeling transcriptomic data to selection and growing strategies. This project ensures that not only ornamental plants make cities look attractive but also improve the local climate, reduce pollution, and improve mental health (Li et al., 2022). Transcriptomics is thus a bridge between sophisticated molecular biology and the capacity of plants to perform well in our day to day dwelling environments.

## 8 Challenges and future perspectives

Beyond calls for better genomes, we prioritize (1) chromosome-scale references for at least one flagship cultivar per genus to stabilize isoform/TSS annotation; (2) paired multi-stress designs (heat×drought×salinity) that reflect urban/horticultural reality rather than single-stress assays; (3) cell-resolved atlases of roots, guard cells, and petals to map aquaporin and ion-exclusion circuits; (4) standardized phenotyping (ionomics, chlorophyll fluorescence, cuticular conductance) to benchmark ‘omics-derived candidates; (5) rapid functional screens (CRISPRi/a, VIGS) to validate hub TFs in the native ornamental; and (6) breeding pipelines that combine a minimal marker set (*SOS/HKT/NHX, P5CS*, selected *bZIP/NAC* hubs) with quality traits so resilience does not compromise ornamentation. This constrains subsequent tasks such as transcriptome assembly, gene annotation and comparative genomics (Deng et al., 2021). Due to this fact, researchers tend to rely on assemblies done by *de novo* that, despite being useful, could overlook minor variations or regulatory fragments that are critical in stress adaptation (He et al., 2019). Due to this, it is highly important to create good reference genomes. Chromosome-scale assemblies generated with long-read sequencing and scaffolding technologies (e.g., Hi-C, optical mapping) would provide more robust support for transcriptomic studies. Such resources would assist in better finding and annotation of genes. They would also assist in locating species-specific transcription anatomy, regulatory anatomy, and structural variants in connection with ornamental qualities (Lu et al., 2021). Opportunities are available with new technologies. The details of cell-level reactions to stress can be documented by single-cell and spatial transcriptomics. This demonstrates how the various cell types within a flower, leaf or root sense and respond to environmental signals (Zhang et al., 2021b). Experiments in model plants have begun to demonstrate the potential of early identification of cell-specific regulatory centers and stress memory-something that would transform the ornamentals with their complex floral structures. The other direction is systems biology ways. Integrating transcriptomic information with metabolomics, proteomics and high-throughput phenotyping may provide full perspectives of stress tolerance. An illustration of this is connecting gene expression to accumulation of metabolites or physiological characteristics that will assist in the discovery of biomarkers and molecular signatures. They can help breeders to select stress-resilient varieties (Li et al., 2022; Wu et al., 2023). Integration of GWAS with transcriptomics has successfully identified regulators of complex traits in crops (e.g., *OsNLP6* for nitrogen use efficiency in rice; Xin et al. (2025). Similar strategies can be extended to ornamentals to dissect polygenic salt and drought tolerance traits.

Lastly, the impacts of climate change do matter. Ornamentals are commonly subjected to a variety of stresses, and heat, drought, and salinity combine in odd combinations. The challenge of designing transcriptomic experiments to recapitulate such combinations of stresses rather than each individual one will be central to deriving helpful results (Saleem et al., 2025). These approaches will ensure that the ornamental breeding keeps abreast with the changes in the environment to defend the beauty and significance of this plant region. In addition to molecular breeding, microbiome-based strategies are emerging as complementary tools for improving stress resilience. For example, *Bacillus subtilis* inoculation enhanced cotton yield, nutrient uptake, and water use efficiency under drought stress by reshaping rhizosphere microbial communities (Ren et al., 2025). Similar microbial interventions could be explored for ornamentals to enhance both resilience and growth performance in water-limited environments. Taken together, these priorities define a forward-looking roadmap: from genomes to cells, from controlled experiments to real-world multi-stress scenarios, and from candidate discovery to applied breeding. By integrating these layers, ornamental crops can be future proofed against climate uncertainty while retaining the aesthetic qualities that underpin their cultural and economic value.

## 9 Conclusion

Together, recent transcriptomes converge on a systems view: early ionic/osmotic sensing, conserved kinase relays, and a small set of TF hubs allocate resources to ions, water, redox, osmolytes, and the protective surface-while species-specific programs preserve color, form, and longevity. The critical shift is from listing DEGs to validating regulons and wiring rules that repeatedly predict field performance. With cell-resolved and multi-stress designs, plus rapid functional genomics and targeted editing, ornamentals can gain durable salt–drought tolerance without aesthetic penalties. The path forward is a compact marker/edit set guided by network context, tested against standardized phenotypes, and deployed in breeding to future-proof urban and commercial horticulture.
